# Ophiobolin O Isolated from *Aspergillus ustus* Induces G1 Arrest of MCF-7 Cells through Interaction with AKT/GSK3β/Cyclin D1 Signaling

**DOI:** 10.3390/md13010431

**Published:** 2015-01-16

**Authors:** Cuiting Lv, Wenxing Qin, Tonghan Zhu, Shanjian Wei, Kui Hong, Weiming Zhu, Ruohua Chen, Caiguo Huang

**Affiliations:** 1 Department of Biochemistry and Molecular Biology, College of Basic Medical Second Military Medical University, 800 Xiangyin Road, Shanghai 200433, China; E-Mails: lvcuiting961021@126.com (C.L.); sjwei8012@hotmail.com (S.W.); 2 Teaching Management Department, Yangpu Hospital, Tongji University School of Medicine, 450 Tengyue Road, Shanghai 200090, China; E-Mail: wendy_yes1@sina.com; 3 Key Laboratory of Marine Drugs, Ministry of Education of China, School of Medicine and Pharmacy, Ocean University of China, Qingdao 266003, China; E-Mail: sdueduzth@126.com; 4 Key Laboratory of Combinatorial Biosynthesis and Drug Discovery, Ministry of Education, School of Pharmaceutical Sciences, Wuhan University, Wuhan 430071, China; 5 VIP Medicine Department, Changhai Hospital, Shanghai 200433, China

**Keywords:** ophiobolin O, G1 arrest, AKT/GSK3β/cyclin D1 signaling

## Abstract

Ophiobolin O is a member of ophiobolin family, which has been proved to be a potent anti-tumor drug candidate for human breast cancer. However, the anti-tumor effect and the mechanism of ophiobolin O remain unclear. In this study, we further verified ophiobolin O-induced G1 phase arrest in human breast cancer MCF-7 cells, and found that ophiobolin O reduced the phosphorylation level of AKT and GSK3β, and induced down-regulation of cyclin D1. The inverse docking (INVDOCK) analysis indicated that ophiobolin O could bind to GSK3β, and GSK3β knockdown abolished cyclin D1 degradation and G1 phase arrest. Pre-treatment with phosphatase inhibitor sodium or thovanadate halted dephosphorylation of AKT and GSK3β, and blocked ophiobolin O-induced G1 phase arrest. These data suggest that ophiobolin O may induce G1 arrest in MCF-7 cells through interaction with AKT/GSK3β/cyclin D1 signaling. *In vivo*, ophiobolin O suppressed tumor growth and showed little toxicity in mouse xenograft models. Overall, these findings provide theoretical basis for the therapeutic use of ophiobolin O.

## 1. Introduction

**Figure 1:**
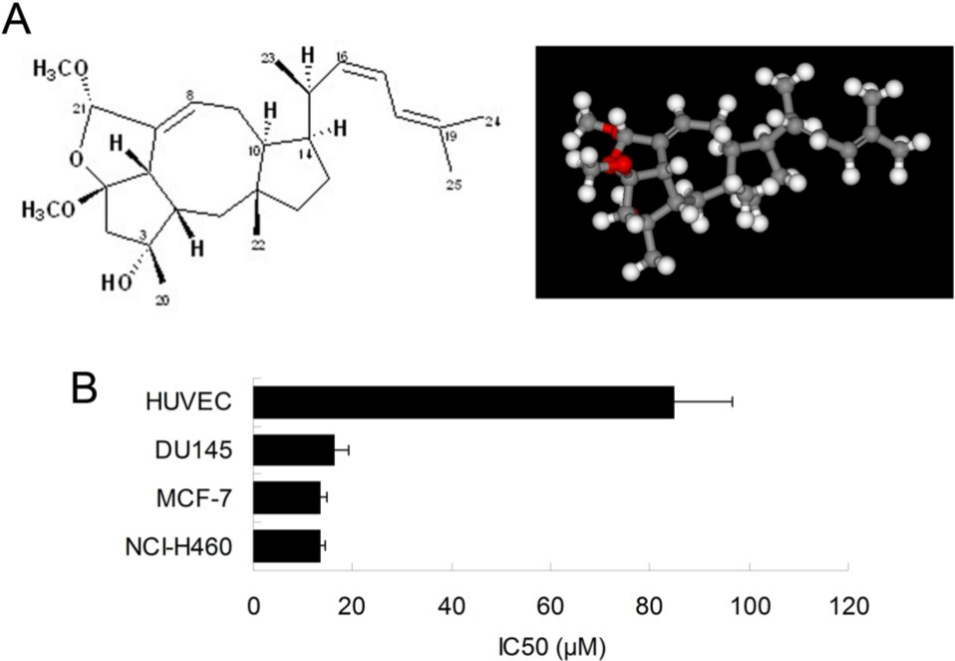
Inhibitory effect of ophiobolin O in human cancer cells. (**A**) Chemical structure of ophiobolin O (**left**); and energy-minimized 3D structure of ophiobolin O (**right**); (**B**) IC_50_ of ophiobolin O on viability of HUVEC, DU145 MCF-7 and NCI-H460cell lines. Cells were treated with ophiobolin O atvarious concentrations for 48 h and processed for MTT assay.

Ophiobolins belong to the family of natural sesquiterpenes that are characterized by a tricyclic (5-7-5) ring system. Ophiobolins as secondary metabolites that are produced by pathogenic fungi, show a broad spectrum of inhibitory activity against nematodes, fungi, bacteria and cancer cells [1,2,3]. Ophiobolin O (Figure 1A) is the member of ophiobolin family, which we have proved is a potent anti-tumor drug candidate for human breast cancer. The ophiobolin O we used is a natural compound that have been isolated from *Aspergillus ustus* 094102 [4].

In previous study, we found that the low concentration of ophiobolin O (0.1 μM) down-regulated the expression of resistance-related protein P-Glycoprotein (P-gp, also known as MDR1), which makes Adriamycin-resistant human breast carcinoma (MCF-7/ADR) cells more sensitive to Adriamycin treatment. Therefore, we demonstrated that ophiobolin O reversed Adriamycin resistance, and suggested ophiobolin O acted as a potential agent to reverse chemotherapy drug resistance [5]. On the other hand, ophiobolin O reduced the viability of human breast cancer MCF-7 cells in dose-dependent manner and efficiently induced apoptosis and cell cycle arrest. We further proved ophiobolin O-induced cell apoptosis was regulated via activation of MAPK signaling pathways. However, the anti-tumor effect and the mechanism of ophiobolin O remain unclear, since we just used one method to detect G1 phase arrest, which lacking of test and verification; and we also did not figure out about the mechanism of ophiobolin O-induced cell cycle arrest at that time [4].

In the current study, we further verified ophiobolin O-induced G1 phase arrest in MCF-7 cells. Then we investigated the putative protein targets of ophiobolin O using INVDOCK program. Three apoptotic or G1 phase arrest-related proteins were extracted. Two of them were closely related to cell apoptosis, and could validate our previous study [4]. The rest one was GSK3β, which is an upstream regulator of G1 checkpoint [6]. In order to check whether GSK3β is involved in ophiobolin O-induced G1 phase arrest, we detected the protein level and knocked down GSK3β expression using siRNA. Then we found that GSK3β knocked-down cells were not sensitive to ophiobolin O anymore, suggesting that ophiobolin O may target GSK3β to induced G1 phase arrest and growth inhibition in MCF-7 cells. Furthermore, ophiobolin O also induced dephosphorylation of AKT, and pre-treatment with phosphatase inhibitor sodium orthovanadate halted dephosphorylation of AKT and GSK3β, and blocked ophiobolin O-induced G1 phase arrest. Therefore, the anti-proliferative effect of ophiobolin O might be linked with G1 to S phase arrest, which was mediated by the Akt/GSK3β/cyclin D1 pathway. Finally, ophiobolin O could suppress tumorigenesis in the xenograft mouse model, suggesting that ophiobolin O suppresses tumor growth in breast cancer.

## 2. Results and Discussion

### 2.1. Ophiobolin O Inhibits the Proliferation of MCF-7 Cells

We have previously shown that the natural compound ophiobolin O isolated from *Aspergillus ustus* 094102 inhibited the growth of human breast cancer cells [4]. In this study, we further examined the inhibitory effect of ophiobolin O on proliferation of other cell lines, such as HUVEC (Human umbilical vein endothelial cell line), DU145 (Human prostate cancer cell line) and NCI-H460 (Human large cell lung carcinoma cell line). The results demonstrated that breast cancer cells MCF-7 was significantly sensitive to ophiobolin O, with IC_50_ value of 13.45 ± 1.26 μM. The IC_50_ values were 84.96 ± 11.73 μM, 16.48 ± 2.68 μM and 13.55 ± 1.01 μM when ophiobolin O treated HUVEC, DU145 and NCI-H460 cells, respectively. It is worth noting that normal human umbilical vein endothelial cells (HUVEC) are less sensitive to ophiobolin O compared with other cancer cell lines (Figure 1B), suggesting that ophiobolin O has a potent anti-cancer activity that preferentially kills cancer cells, especially breast cancer cells, over normal cells. This selectivity led us to question the mechanism by which ophiobolin O exerted its effect on MCF-7 cells.

### 2.2. Ophiobolin O Induces G1 Arrest in MCF-7 Cells

**Figure 2:**
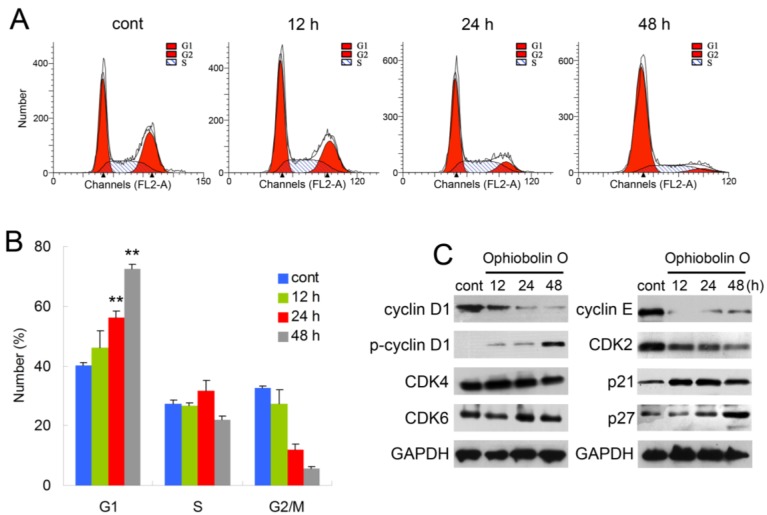
Ophiobolin O induces cell cycle arrest in MCF-7 cells. (**A**) Ophiobolin O caused cell cycle arrest at the G1 phase. Cells were treated with vehicle and 15 μM Ophiobolin O for 12, 24, and 48 h, and then cell cycle distribution was assessed using flow cytometry; (**B**) The percentage of cells in different phases of the cell cycle was represented by a bar diagram. The percentage of cells in each population was shown as mean ± SD from three independent experiments. * *p* < 0.05, ** *p* < 0.01; (**C**) Western blot analysis of G1 transition-related proteins were analyzed by Western blotting assay. GAPDH was used as an equal loading control.

In order to test whether the inhibition effect of ophiobolin O was related to cell cycle progression, cell cycle distribution and related checkpoint factors were studied. MCF-7 cells were treated with 15 μM ophiobolin O for 12 h, 24 h and 48 h, respectively. The results showed that cells treated with ophiobolin O accumulated progressively in G1 phase (Figure 2A,B). Compared with the negative control, treatment with ophiobolin O resulted in a significant increase in the proportion of G1 phase cells (control: 40.19% ± 1.03%; 12 h: 46.09% ± 5.79%; 24 h: 56.18% ± 2.19%; 48 h: 72.56% ± 1.35%) and about a 1.8-fold increase after 48 h. Meanwhile, G2/M reduced appreciably from 32.57% ± 0.69% to 5.53% ± 0.88% during 48 h incubation. These results suggest that G1 phase arrest accounts for the anti-proliferative effect of ophiobolin O observed in MCF-7 cells. Besides, ophiobolin O treatment resulted in a time-dependent decrease in the protein expression of cyclin D1, cyclin E, CDK2 and increase of p-cyclin D1 (Thr286), p21 and p27. However, the protein levels of CDK4 and CDK6 were not significantly changed during ophiobolin O treatment (Figure 2b).

### 2.3. Putative Protein Targets for Ophiobolin O

**Figure 3:**
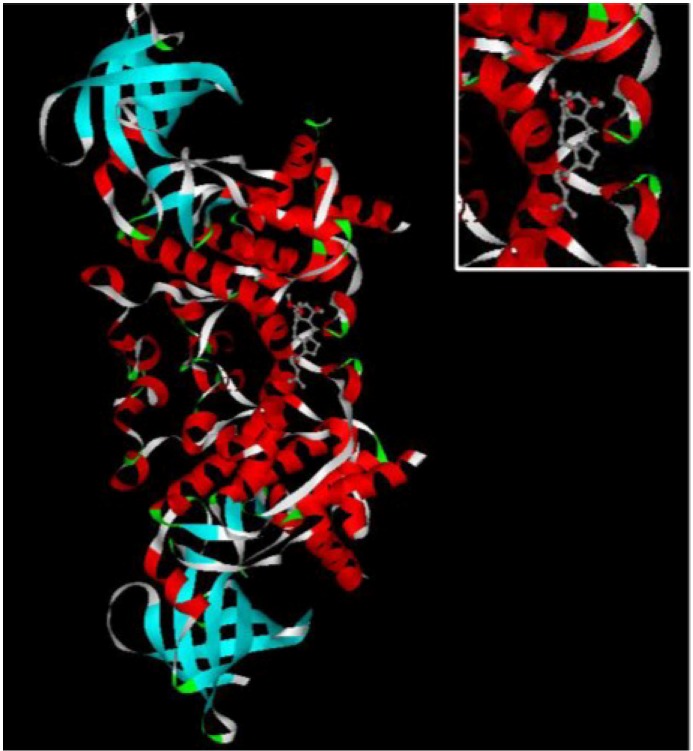
The INVDOCK analysis of ophiobolin O binds to GSK3β. Illustration of the ophiobolin O molecule docked into the GSK3β protein, modeled using the INVDOCK program; upright: the Amplification of binding area.

Previously, we observed that ophiobolin O-induced cell apoptosis was regulated via activation of MAPK signaling pathways in MCF-7 cells; but for cell cycle arrest, we did not elucidate how the ophiobolin O induced G1 phase arrest. Furthermore, to establish the anti-cancer mechanism of ophiobolin O, an inverse docking (INVDOCK) analysis was applied to identify the possible targets of ophiobolin O. Using the INVDOCK program, 90 putative proteins were extracted from the Protein Data Bank. Of these, three proteins were closely related to apoptosis and G1 phase arrest (Table 1). TNF (Tumor necrosis factor) is related to cell apoptosis [7]; MAP2K1 (Dual specificity mitogen-activated protein kinase kinase 1; ERK), which is a member of MAPKs, has been proved to be activated during the ophiobolin O-induced apoptosis in MCF-7 cells [4]; GSK3β (Glycogen synthase kinase-3 beta)regulates G1 phase transition through Phosphorylation of cyclin D1 which is one of the key regulatory proteins controlling the transition fromG1 to S phase [8], therefore, in order to study the mechanism of ophiobolin O-induced G1 phase arrest, 3-D structure of GSK3β was chosen to explore its binding interaction with ophiobolin O. The illustration of ophiobolin O docked to GSK3β using the INVDOCK program is shown in Figure 3. Such an analysis indicated that ophiobolin O could form a reasonable drug-protein complex with GSK3β, which provided a hypothesis that ophiobolin O may induce G1 arrest of MCF-7 cells through interaction with GSK3β/cyclin D1 signaling.

**Table 1:** Apoptotic or G1 phase-related proteins predicted by INVDOCK to bind to ophiobolin O.

Compond	Protein	Gene	Gene ID	Species	Ligand-Protein Interaction Energy Value
Ophiobolin O	Glycogen synthase kinase-3beta	GSK-3β	2932	Human	−35.8
	Tumor necrosis factor	TNF	7124	Human	−37.5
	Dual specificity mitogen-activated protein kinase kinase 1	MAP2K1	5604	Human	−37.6

### 2.4. Ophiobolin O Induces G1 Arrest of MCF-7 Cells through Interaction with AKT/GSK3β/Cyclin D1 Signaling

**Figure 4:**
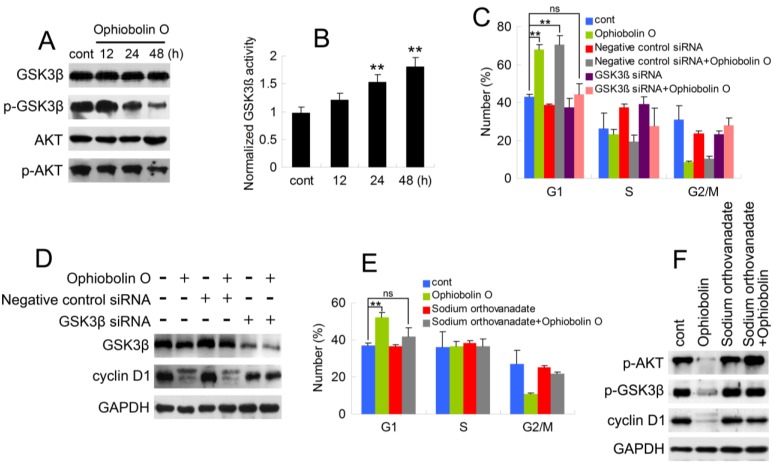
AKT**/**GSK3β signaling is involved in ophiobolin O-induced G1 phase arrest in MCF-7 cells. (**A**) GSK3β, p-GSK3β (Ser 9), AKT and p-AKT (Ser 473) were detected after ophiobolin O-treatment for 48h by western blotting analysis; (**B**) Statistical results showing the normalized enzyme activities of GSK3β. Cells were treated with 15 μM ophiobolin O, harvested at 12 h, 24 h and 48 h. GSK3β activity was determined using GSK3β enzyme activity detection kit, and normalized *versus* control group; (**C**) Cells transfected with GSK3β siRNA or not were treated with or without 15 μM ophiobolin O for 48 h, then harvested for cell cycle analysis or (**D**) western blotting assay against GSK3β and cyclin D1. GAPDH was used to ensure equal protein loading; (**E**) Cells pre-treated with sodium orthovanadate or not were incubated with or without 15 μM ophiobolin O for 48 h, then harvested for cell cycle analysis or (**F**) western blotting assay against p-AKT, p-GSK3β and cyclin D1. Values were means ± SD of three independent experiments. * *p* < 0.05, ** *p* < 0.01 *versus* control group.

As described above, it is essential to check whether GSK3β is involved in ophiobolin O-induced G1 phase arrest. Therefore, protein level of GSK3β was analyzed by Western blotting. Furthermore, consider that Akt/GSK3β signaling plays a critical role in cyclin D1 expression and carcinogenesis, we also examined AKT levels. As shown in Figure 4A, ophiobolin O treatment reduced the phosphorylation level of Akt (Ser 473) and GSK3β (Ser 9), indicating an increased activity of GSK3β. And GSK3β activity assay proved the increased enzyme activity of GSK3β *in vitro* (Figure 4B). Furthermore, we used siRNA to knock-down GSK3β, and observed that cells transfected with GSK3β siRNA were not sensitive to ophiobolin O anymore: ophiobolin O-induced G1 phase arrest in MCF-7 cells was significantly abolished; and the decrease of cyclin D1 expression was brought to normal levels (Figure 4C,D). Finally, phosphatase inhibitor sodium orthovanadate was applied to halt dephosphorylation of AKT and GSK3β, and the pre-treatment also blocked ophiobolin O-induced G1 phase arrest and cyclin D1 reduction.

Taken together, these results suggest that ophiobolin O treatment reduces the phosphorylation of Akt and subsequent phosphorylation of GSK3β, indicating Akt inactivation and GSK3β activation respectively. This might contribute to cyclinD1 degradation and G1-phase arrest, cause GSK3βis a critical regulator of cyclin D1 expression [6,9,10].

### 2.5. Ophiobolin O Inhibits Tumor Xenograft Growth

**Figure 5:**
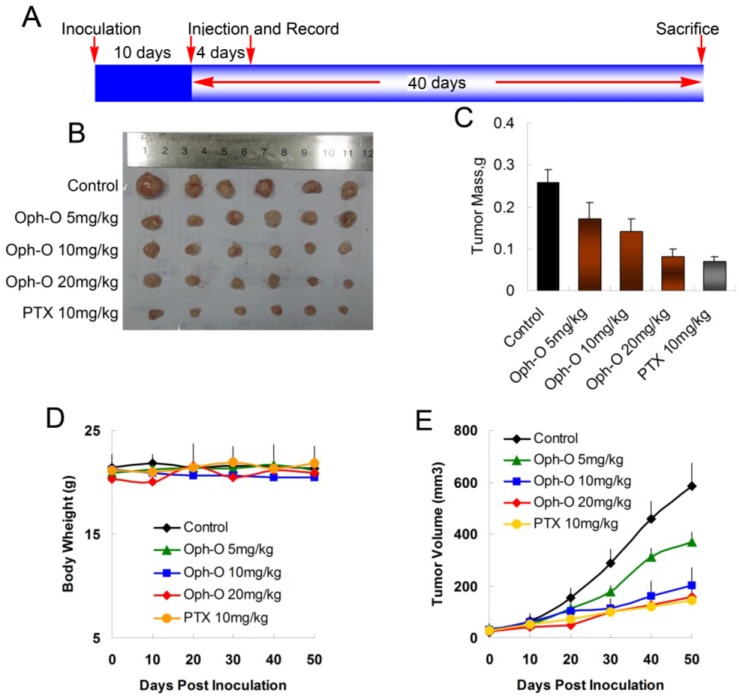
Effect of ophiobolin O on tumor growth. (**A**) BALB/c male athymic mice were injected 5 × 10^6^ MCF-7 cells s.c. for the development of subcutaneous tumors. The mice were randomized into five groups and treated with Vehicle (1% DMSO), 10 mg/kg PTX or various concentrations of ophiobolin O (5, 10, or 20 mg/kg) i.v. every four days until sacrifice according to the protocol in panel (**A**); (**B**) Tumor image from various treatment groups; (**C**) Average tumor mass at sacrifice; (**D**) Tumor volume measurements; (**E**) Body weight during the treatment.

To evaluate whether ophiobolin O inhibits tumor growth *in vivo*, MCF-7 cells were injected s.c. into the right armpit of six-week old BALB/c female athymic mice. The injected carcinoma cells grew into palpable tumors in the nude mice within 10 days (Figure 5A). Paclitaxel (PTX)-treated mice (10 mg/kg) were used as positive control to assess the effect and toxicity of ophiobolin O. In this study, the tumor growth in mice treated with ophiobolin O (5, 10, or 20 mg/kg/day) was dose-dependently slowed (Figure 5B,C). Tumor volume was significantly reduced during ophiobolin O treatment (Figure 5E). The inhibitory rates at 50th day of 5, 10, and 20 mg/kg ophiobolin O treatment group were 34.62%, 46.15%, and 69.23%, respectively. The 20 mg/kg ophiobolin O treatment showed nearly equivalent effect compared to positive control (inhibition rate of 73.08%). Furthermore, no significant weight loss was observed during the course of ophiobolin O treatment, indicating there is feasibility for upward adjustment of dosage for improved treatment outcomes (Figure 5D).

### 2.6. Discussion

So far, studies have shown that ophiobolin O significantly suppressed tumor growth *in vivo* and *in vitro*. It triggered cell cycle arrest at G1 phase through interaction with AKT/GSK3β/cyclin D1 signaling.

In our obvious studies, significant attention was focused on the apoptosis induced by ophiobolin O, and the important role of MAPK signaling pathway involved in apoptotic death in MCF-7 cells. MAPK signaling cascades have been proved to play essential roles in the regulation of a wide variety of cellular processes [11], and several key signaling components and phosphorylation events participate in regulating the cell cycle, apoptosis and even tumorigenesis [12,13]. JNK, ERK and p38 are three members of the MAPK family [14]. The JNK pathway is considered to be responsible for the apoptotic response induced by several anti-tumor agents [15], and we have reported that the activation of JNK induced by ophiobolin O triggered apoptosis through the phosphorylation of Bcl-2 in MCF-7 cells. Otherwise, ERK and p38 activation were also observed during ophiobolin O treatment. The p38^SAPK2^ and ERK2 have been shown to regulate G1 transition through interaction with cyclin D1 stability by phosphorylating T286 in some reports [16,17,18]. However, here we declare that AKT/GSK3β/cyclin D1 signaling is responsible for ophiobolin O-induced cyclin D1 degradation and G1 arrest.

In tumor cells, the abnormal molecular activity directly induces the uncontrolled cell proliferation, which is one of the hallmarks of cancer. The majority of human cancers have been reported to have alterations in the function of cell cycle regulatory proteins [19,20,21]. Cyclin D1 and cyclin E are the key regulatory proteins controlling the transition from G1 to S phase by binding to Cdk4/Cdk6 and CDK2 [12]. Considering the crucial role of cyclin D1, it is not surprising that its expression is down-regulated in cells stimulated with anti-proliferative cytokines. This down-regulation generally manifests at the level of protein stabilization, which was accelerated via the phosphatidylinositol 3-kinase Akt/GSK3β pathway [9]. In normal cells, the levels of cyclin D1 begin to rise early in G1 phase and continue to accumulate until the G1/S-phase boundary when levels rapidly decline [13,14]; glycogen synthase kinase 3β (GSK3β) induces rapid proteolysis of cyclin D1 by phosphorylating cyclin D1 on threonine residue 286 (Thr 286) [8]. Phosphorylation facilitates cyclin D1 nuclear export by the exportin-chromosome maintenance region 1 (CRM1), and the phosphorylated form of the cyclin is subsequently degraded within the cytoplasm [22,23,24]. In this manner, GSK3β regulates nuclear export and stability of cyclin D1 to ensure the normal mitosis. Usually, GSK3β has a higher level of basal activity in tumor cells than in their normal counterparts [25]. Thus, the inactivation of GSK3β and subsequent up-regulation of cyclin D1 may have a critical role in human cancer cells. In these cells, phosphorylation at Ser 9 by AKT is a major means to inactivate GSK3β [26,27].

In our study, no obvious amount of changes of total GSK3β were observed during the treatment. In contrast, numerous phosphorylation form of GSK3β (Ser 9) was detected in control cells, and showed marked time-dependent decrease after ophiobolin O treatment (Figure 4A), consistently with increase of GSK3β activity (Figure 4B). Therefore, the expression of cyclin D1 reduced and phosphorylated cyclin D1 on threonine residue 286 was subsequently increased (Figure 2C). These results indicated that the inactivation of GSK3β contributes to MCF-7 cell proliferation, and GSK3β/cyclin D1 cell signaling maybe involved in ophiobolin O-induced G1 phase arrest. The INVDOCK was designed to confirm the potential targets related with ophiobolin O-induced anti-neoplastic effect, and the results noted that ophiobolin O could directly bind to GSK3β (Table 1 and Figure 3). It is accepted that small-molecule drugs generally exert their therapeutic functions by binding to the cavities of proteins to influence their biological activities [28]. This binding may influence phosphorylation of GSK3β, therefore activate GSK3β and down-regulate cyclin D1 to induce G1 phase arrest. To investigate weather the putative protein target-GSK3β is responsible for ophiobolin O-induced cyclin D1 degradation and G1 arrest, we used siRNA to knock-down GSK3β, and noticed ophiobolin O-induced reduction of cyclin D1 and G1 arrest were significantly abolished (Figure 4C–E). Otherwise, considering GSK3β as a Akt-inactivating factor, we also detected the AKT effect during ophiobolin O treatment. It appeared that ophiobolin O inhibited the phosphorylation level of Akt at Ser 473. Furthermore, we used the sodium orthovanadate to up-regulate Akt activity. The sodium orthovanadate is an inhibitor of tyrosine phosphatases, alkaline phosphatases and a number of ATPases; commonly used to improve the activation of protein by inhibiting the dephosphorylation or promoting phosphorylation. It prevents decreased Akt-Ser-473 phosphorylation in the CA1 region, which up-regulates Akt activity [29]. In our study, sodium orthovanadate was applied to halt dephosphorylation of AKT and GSK3β, and the pre-treatment also blocked ophiobolin O-induced G1 phase arrest and cyclin D1 reduction. Therefore, we conclude that Ophiobolin O induces G1 arrest of MCF-7 cells through interaction with AKT/GSK3β/cyclin D1 signaling.

**Figure 6:**
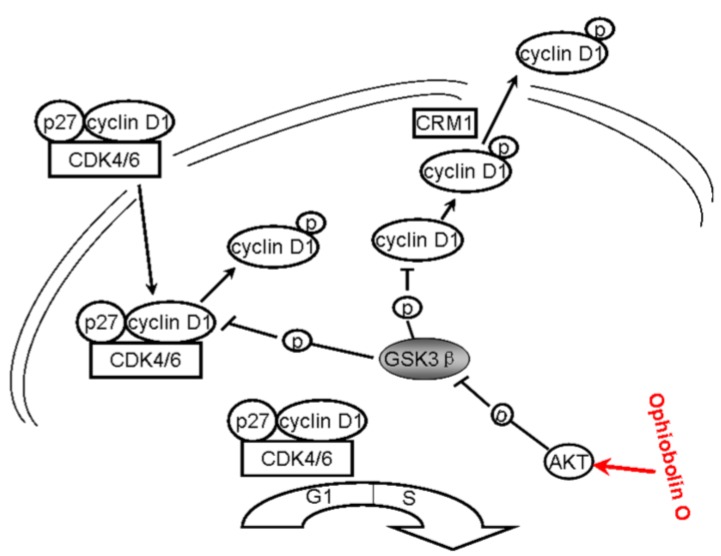
A schematic demonstration for ophiobolin O inducing G1 arrest in MCF-7 cells through interaction with AKT/GSK3β/cyclin D1 signaling.

All above strongly suggest that ophiobolin O may target AKT/GSK3β/cyclin D1 signaling. The increase of active form of GSK3β accelerates cyclin D1 degradation, and inventually induces G1 phase arrest in MCF-7 cells. Cyclin D1 regulation and the probable mechanisms of ophiobolin O-induced G1 phase arrest are characterized in Figure 6.

## 3. Experimental Section

### 3.1. Reagents

DMEM, fetal bovine serum, penicillin and streptomycin were purchased from Biowest (Maine et Loire, France). Cell cycle detection kits were provided by Nanjing KeyGen Biotech Co. Ltd. (Nanjing, China). GAPDH was obtained from Tianjin Sungene Biotech (Tianjin, China). Antibodies against cyclin D1 (#2926), p-cyclin D1 (Thr 286, #3300), GSK3β (#9315) p-GSK3β (Ser 9, #4688), CDK4 (#2906) and CDK6 (#3136) were purchased from Cell Signaling Technology (Beverly, MA, USA). siRNA used for silence GSK3β gene was purchased from Shanghai Gene Pharma Co., Ltd (Shanghai, China).

### 3.2. Chemical Preparation and Cell Culture

Ophiobolin O is a natural compound that has been isolated from *Aspergillus ustus* 094102. The compound was dissolved in 100% DMSO and stored at −20 °C, then diluted with cell culture media before using. The final DMSO concentration did not exceed 0.1%. Human breast cancer cell line MCF-7 was purchased from the cell bank of the Shanghai Institute of Cell Biology (Shanghai, China),and was cultured in DMEM with 10% fetal bovine serum and antibiotics (100 μg/mL streptomycin and 100 U/mL penicillin) in a humidified 5% CO_2_ incubator at 37 °C.

### 3.3. Cell Viability Assay

Cells were seeded into 96-well plates at 4 × 104 cells/mL, incubated for 24 h, and then treated with the indicated concentrations of ophiobolin O (0, 2.5, 5, 10, 20, 40 and 80 μM) for 48 h. Cell viability was determined using MTT assay.

### 3.4. Cell Cycle Analysis

Cells were harvested, washed twice with PBS, fixed in 70% ethanol and stored at 4 °C overnight, then washed with PBS, incubated with RNase at 37 °C for 30 min, and stained with PI (1 mg/mL) in the presence of RNase A for 30 min. Cell cycle phase analysis was performed by using a FACScalibur flow cytometer.

### 3.5. Western Blot Analysis

Cells were lysed in Western blotting lysis buffer (50 mM Tris, 150 mM NaCl, 1% Triton X-100, 1% sodium deoxycholate, 0.1% SDS and 1 mM PMSF) at 4 °C for 30 min. After 12,000× *g* centrifugation for 15 min, the protein content of supernatant was quantified using BCA protein assay kit (Beyotime, Shanghai, China). Equal amounts of the protein samples were separated by SDS-PAGE, and transferred to nitrocellulose membranes using an electro-blotting apparatus (Bio-Rad, Hercules, CA, USA). Then the membranes were blocked in blocking buffer (TBST plus 5% skimmed milk), and incubated with primary antibodies overnight at 4 °C. After that the membranes were washed with TBST and incubated with HRP (horseradish peroxidase)-conjugated secondary antibodies for 1.5 h at 4 °C. Protein expression was visualized using the chemoluminescence reagent (Millipore, Billerica, MA, USA) and detected on photographic film.

### 3.6. Enzyme Activity Assay

Activity of GSK3β was determined by the GSK3β enzyme activity detection kit (GENMED, Shanghai, China) according to manufacturer’s instructions. Briefly, the samples of drug-treated cells were rinsed by reagent A and then re-suspended with the extraction buffer reagent B. After 30 min incubation on ice, the mixture was centrifuged at 4 °C. The supernatant was collected and then assayed for enzyme activity, by mixing 65 μL reagent C, 10 μL reagent D, 10 μL reagent E and 10 μL reagent F and incubation at 30 °C for 3 min. Immediately after the addition of 5 μL supernatant to the reagent mixture, the optical density was measured at 340 nm using a Microplate Reader at 0 min and 5 min. The activity was measured as the difference between the absorbance value at 0 and 5 min [30].

### 3.7. Identification of Putative Protein Targets for Ophiobolin O

To verify the proteins related to possible Ophiobolin O targets, a flexible ligand-protein inverse docking program, INVDOCK was used to identify putative protein targets for Ophiobolin O. The 3D structure of Ophiobolin O was input into the INVDOCK program. The software automatically searched for protein cavities derived from 3D structures of all candidate proteins. An energy value statistically derived from the analysis of a large number of PDB ligand-protein complexes was used as a threshold for screening likely binders. A human protein was considered as a putative target of Ophiobolin O if the molecule could be docked into the protein and the binding satisfied a molecular-mechanics based criterion for chemical complementarity [31].

### 3.8. siRNA Transfection

Cells were plated in six-well plates, grown for 24 h, then transfected with 80 pmol of siRNA for 12 h using Lipofectamine 2000 reagent and OPTIMEM reduced serum medium (Invitrogen, Carlsbad, CA, USA). The cells were assayed within 72 h after transfection.

### 3.9. Mouse Xenograft Model

The mouse xenograft model was established by injection of 5 × 10^6^ cells s.c. into the right armpit of five-week old BALB/c female athymic mice (National Rodent Laboratory Animal Resource, Shanghai, China). The mice were randomized into vehicle control and treatment groups when xenografts were palpable after 10 days. Vehicle or drugs were administered i.v. every four days until sacrifice; body weight and tumor size were measured and recorded at the same time. Tumor size was measured using electronic caliper, and the tumor volumes were calculated using the formula: length × width^2^/2. On 50 days, mice were sacrificed; tumors were collected, weighed, and photographed. Tumor inhibition effect was calculated using the following equation: tumor suppression (%) = (1 − T/C) × 100, where T is the average tumor weight of the treated group and C is that of the control group.

## 4. Conclusions

Ophiobolin O induces G1 phase arrest in human breast cancer MCF-7 cells. It targets GSK3β and interacts with GSK3β/cyclin D1 signaling pathway to induced growth inhibition. *In vivo*, ophiobolin O suppressed tumor growth and showed little toxcity in mouse xenograft models.
